# A Systematic Review of Virtual Reality vs. Standard Social Skills Training for Improving Social Interaction Skills and Reducing Social Anxiety in Children With Autism Spectrum Disorder (ASD)

**DOI:** 10.1192/bjo.2024.171

**Published:** 2024-08-01

**Authors:** Oluwatobi Idowu, Nicholas Aderinto, Gbolahan Olatunji, Emmanuel Kokori

**Affiliations:** 1 Cheshire and Wirral Partnership NHS Foundation Trust, Chester, United Kingdom; 2 Ladoke Akintola University of Technology, Ogbomoso, Nigeria; 3 University of Ilorin, Ilorin, Nigeria

## Abstract

**Aims:**

Autism Spectrum Disorder (ASD) poses unique challenges for social interaction and communication skills development in children. Various interventions, including virtual reality (VR) and social skills training, have emerged as potential approaches to address these challenges. This systematic review aims to evaluate and compare the effectiveness of VR Social Skills Training with Standard In-Person Social Skills Training in improving social interaction skills and reducing social anxiety levels in children with ASD.

**Methods:**

A search was conducted across electronic databases (PubMed, PsycINFO, Cochrane Library and Scopus) for relevant studies published from 2000 to December 2023. Inclusion criteria include randomised controlled trials (RCTs) and observational studies comparing VR Social Skills Training with Standard In-Person Social Skills Training in children diagnosed with ASD within the specified age range. Two independent reviewers assessed study eligibility, conducted data extraction, and evaluated study quality. The primary outcomes included changes in social interaction skills and reduced social anxiety levels.

**Results:**

From 1,239 studies initially identified, 25 met inclusion criteria post-screening. VR interventions (n = 12) showed significant improvements (80%) in social interaction skills (15% average anxiety reduction). Varied platforms were utilised, including virtual social scenarios. Using conventional techniques, standard interventions (n = 13) demonstrated improvements (75%) with a 12% average anxiety reduction. Comparative effectiveness between VR and Standard approaches lacked consistent significance. Subgroup analyses showed shorter interventions (4–8 weeks) induced rapid skill improvements, while longer-term ones (12+ weeks) sustained anxiety reduction. Younger participants (6–8 years) exhibited more pronounced skill enhancements and higher baseline anxiety correlated with greater improvement.

**Conclusion:**

This review provides an overview of the current evidence on the comparative effectiveness of VR Social Skills Training and Standard In-Person Social Skills Training for children with ASD. The implications of this review extend to clinicians, educators, and policymakers involved in developing and implementing interventions aimed at improving social outcomes in children with ASD.

